# A tablet-based task for assessing environmental preferences in children and adults

**DOI:** 10.1016/j.mex.2019.08.002

**Published:** 2019-08-23

**Authors:** Kimberly L. Meidenbauer, Cecilia U.D. Stenfors, Michael P. Ingram, Marc G. Berman

**Affiliations:** The University of Chicago, United States

**Keywords:** Environmental preference task, Nature preferences, Child development, Android application

## Abstract

To assess environmental preferences in a wide age range of children, a tablet-based task was developed which can be used with children as young as 4-years-old. The current method uses images which have been rated on aesthetic preference in a separate adult sample to ensure that the nature and urban images shown are matched on aesthetics, allowing for a true evaluation of environmental preferences (without any confound of general aesthetics). However, the app is customizable and can evaluate preference for any set of 10 images researchers may be interested in (i.e. is not limited to environmental preferences or these specific images).

• This task runs as an Android tablet app
• The app allows for up to 10 pictures at a time and uses a ranking of 4 images on each trial (from frowny to happy face) where each image is compared with every other image at least once
• The app was successfully employed with a sample of 4-to-11-year-old children and their parents/guardians

## Graphical abstract

**Figure:**
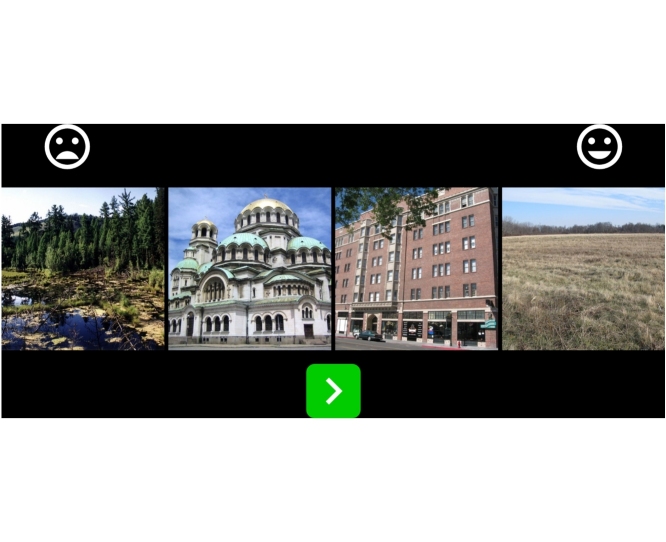

**Specifications Table**

**Table:** 

Subject Area:	Psychology
More specific subject area:	Environmental Psychology
Method name:	Environmental preference task
Name and reference of original method:	Meidenbauer, K. L., Stenfors, C. U. D., Young, J., Layden, E. A., Schertz, K. E., Kardan, O., Decety, J., & Berman, M.G. (2019) The gradual development of the preference for natural environments. Journal of Environmental Psychology, 65. doi.org/10.1016/j.jenvp.2019.101328
Resource availability:	Stimuli & Task code at: https://osf.io/xj3pk/

## Method details

### Overview

Though adult preference for natural environments over urban environments are well documented [[1], [2], [3], [4]], few studies have examined whether these preferences are apparent in children. The current task was designed to evaluate the environmental preferences of children using an app that is appropriate for a wide age range.

This task assesses environmental preferences using a custom-made application which can be installed on a touch-screen tablet running a recent Android OS. It can be used with children as young as 4 years of age. The method was employed by Meidenbauer et al. [5] in a large sample of children aged 4–11 and their parents or guardians.

### Task details

**Fig. 1:**
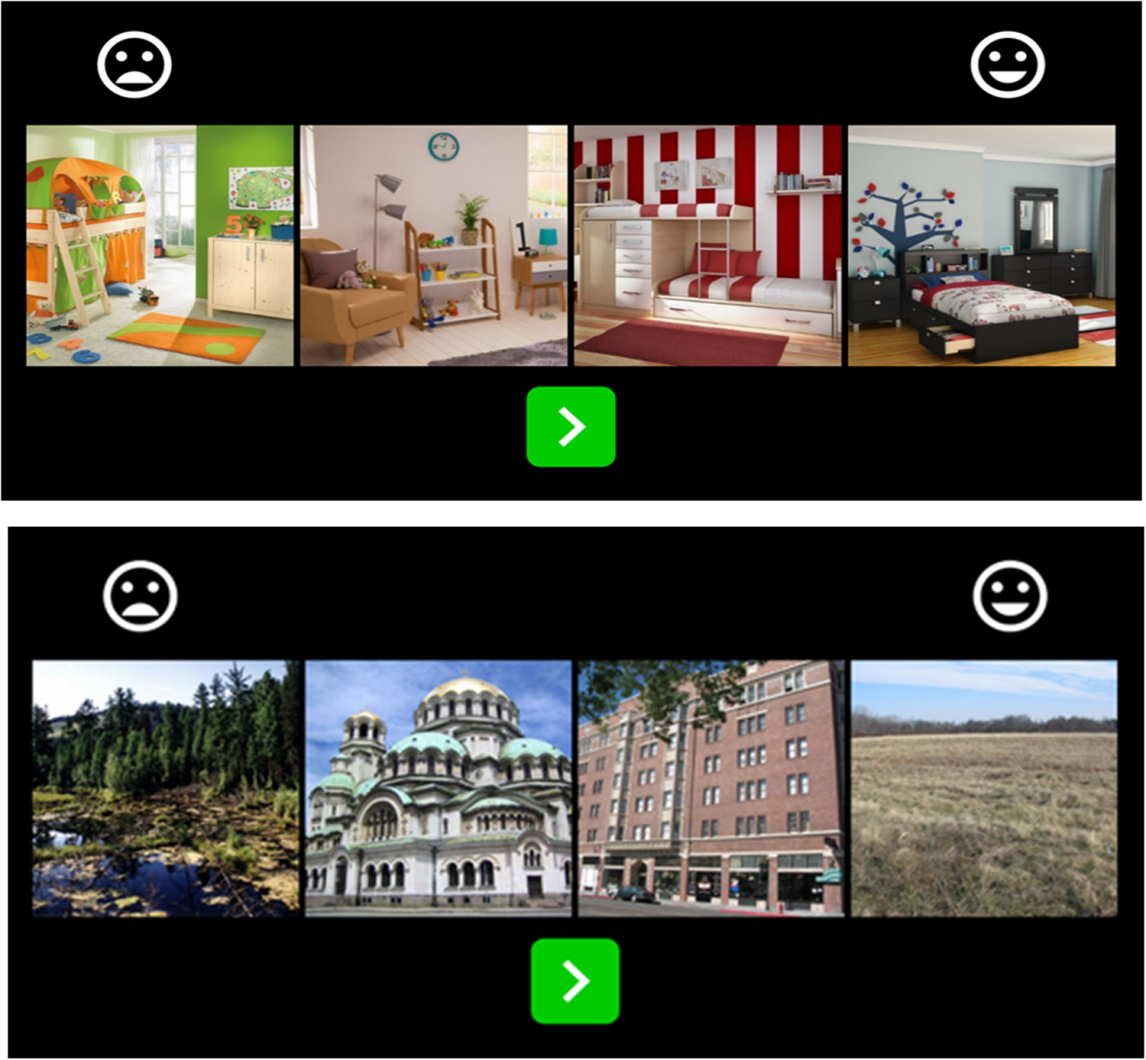
Task Design. Upper panel depicts a trial from the practice rounds. Lower panel depicts a sample trial from the actual experiment, which contains images in the following conditions (from left to right): high aesthetic value nature, high aesthetic value urban, low aesthetic value urban, low aesthetic value nature.

The task was designed for use on a touch-screen tablet, which allows participants to drag the images left and right to put them in the preferred order, where the frowny face indicates the least preferred and the smiley face indicates the most preferred. This comparison approach was used (rather than a Likert-type response scale) as young children in a pilot study (˜4 years old) had difficulty placing the pictures on the happy-to-frowny face scale but were able to say which pictures they liked more than the others. This pilot study showed that the use of Likert-type response scales on singular images for children in the lower end of our age range tended to result in a more binary decision (choosing the anchors of smiley vs. frowny face) rather than along a continuum. The task includes 10 trials where four images are shown at a time. The presentation of images is randomized across trials and across starting positions within a trial. Because of this randomization, any set of four images from the 10-image set can appear in a trial, but the app employs an algorithm to ensure that each of the 10 images were compared to every other image in the set at least once (Fig. 1).

### Android app details

The code for the tablet app and our stimuli can be found at: https://osf.io/xj3pk/. The app includes a practice trial version which orients children to the format of the task using images of children’s bedrooms but does not require a subject number or save the data. There are two versions of the task (IRT_1 and IRT_2) which have different picture sets. For each of these two versions, a subject number must first be entered, then a short demographics page appears for the experimenter to fill out. Here, the experimenter can enter whether the participant is a child or their parent, what is the participant’s gender, and what is their age.

### Procedure

*Instructions for children*: “You are about to see sets of four pictures, and you will be asked to put them in order based on how much you like them. On the one end you will see a frowny face, and on the other you will see a smiley face. I want you to move these pictures around so that the pictures are in order of the one you like the least by the frowny face to the one you like the most by the smiley face. When you’ve put the photos in order of your least to your most favorite, you can press the green button to go onto the next set of pictures.”

Next the child does four practice trials with the experimenter where they are asked to sort images of children’s bedrooms before continuing onto the real task. For the first practice trial, the experimenter moves the bedrooms around on the tablet screen to demonstrate how this is done and reminds the child about the smiley and frowny face anchors before asking them to start.

### Picture stimuli

**Fig. 2:**
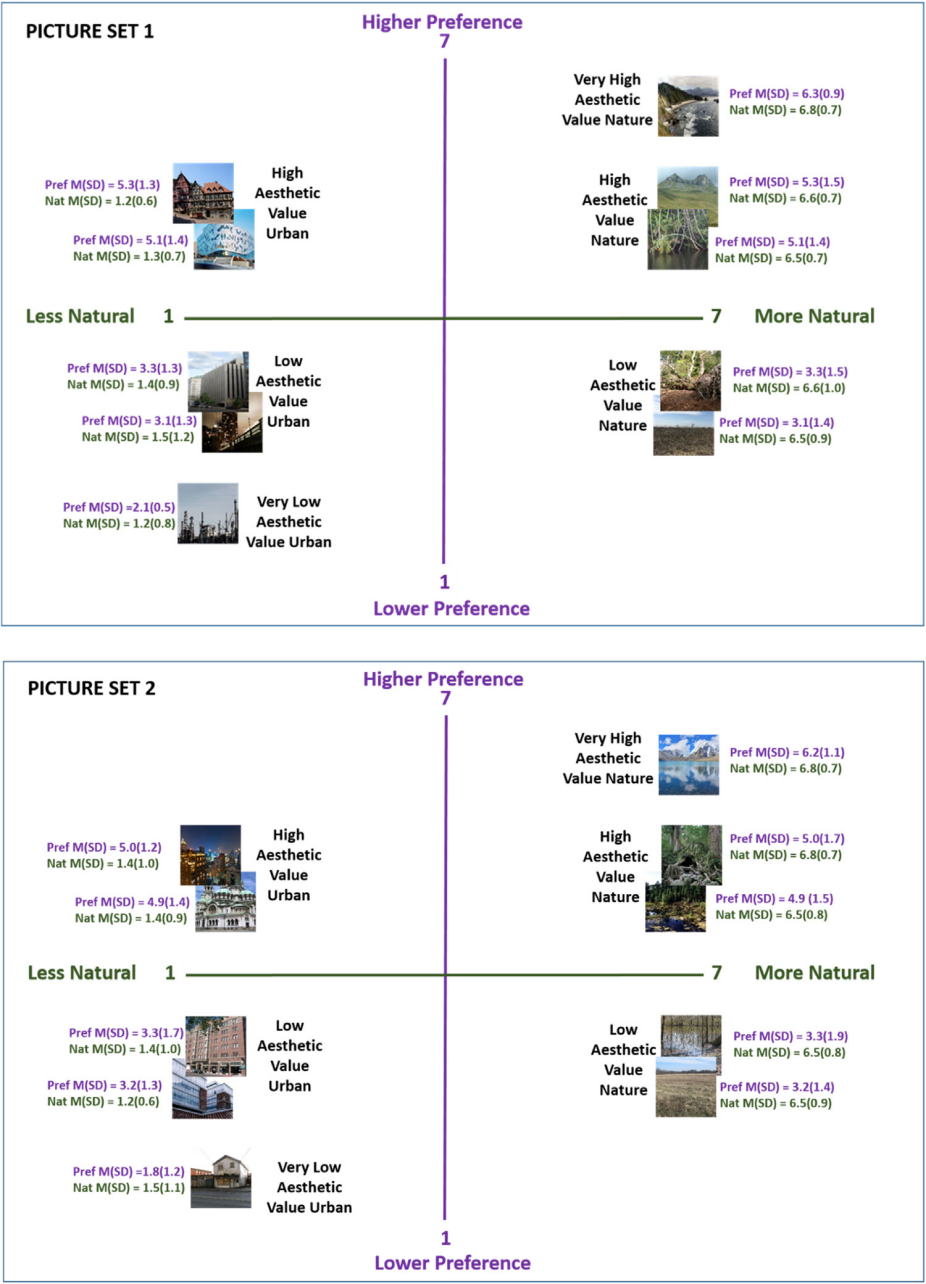
Aesthetic value and Naturalness ratings for Image Sets. Plotting of aesthetic value (Y axis) and naturalness (X axis) of the images in each set used in the current task. Ratings taken from the previous validation study.

The specific stimuli used were taken from an image set which was rated on several attributes (including aesthetic preference and naturalness) in a previous validation study. In this validation study, adult participants rated a set of over 300 nature and urban images on a 1–7 Likert scale (1 = strongly dislike to 7 = strongly like). The preference ratings from this validation study were used to select the particular stimuli for the current study (Table 1). Our goal was to find sets of nature and urban images which were rated very similarly on aesthetic preference to ensure that we’d be able to examine environmental preferences in children and any observed effects would not be attributable simply to differences in aesthetics (Fig. 2. Full sized versions of all stimuli can be found at: https://osf.io/xj3pk/

**Table 1:** Average image aesthetic value pre-ratings for each picture set.

	Very High Aesthetic Value Image	High Aesthetic Value Image #1	High Aesthetic Value Image #2	Low Aesthetic Value Image #1	Low Aesthetic Value Image #2	Very Low Aesthetic Value Image
**Picture Set 1**						
Nature	6.31	5.30	5.12	3.28	3.12	
Urban		5.29	5.11	3.28	3.06	2.09
**Picture Set 2**						
Nature	6.19	5.02	4.86	3.30	3.22	
Urban		5.04	4.88	3.30	3.22	1.77

Ratings on a 1–7 scale (1 = strongly dislike, 7 = strongly like) for images in each picture set. These ratings were gathered from a separate validation study with a normative adult sample. The images in each picture set were chosen with the goal of ensuring that the nature and urban images in the same aesthetic value category (i.e. High Aesthetic Value) were very closely matched on preference.

### Instructions for building and installing the preference task android app

Part I: Build Android app(s) from the source code

1 Download and install Android Studio 3.0.1 on your computer: https://developer.android.com/studio/archive
2 Unzip the *child_irt.zip* file (https://osf.io/hc2ne/) and open the unzipped folder with Android Studio.
3 There are three versions of the app, **Condition 1**, **Condition 2**, and **Practice**. We will build all three versions as separate apps.
  a Build the **Condition 1** version by clicking on **Build Variants** on the left vertical bar of Android Studio. In the window that opens, change the 'Build Variant' to '*c1Debug*.' Then, in the top menu, go to **Build** -> **Build APK(s)** and wait for Android Studio to build the Condition 1 version of the app.
  b Build the **Condition 2** version by selecting the '*c2Debug*' Build Variant. Then, in the top menu, go to **Build** -> **Build APK(s)** and wait for Android Studio to build the Condition 2 version of the app.
  c Build the **Practice** version by selecting the '*practiceDebug*' Build Variant. Then, in the top menu, go to **Build** -> **Build APK(s)** and wait for Android Studio to build the Practice version of the app.
4 Now there should be three folders named c1/, c2/, and practice/ in the folder child-irt/app/build/outputs/apk. Inside each of these three folders, there should be a folder named release/ which contains a file that ends in. apk, such as app-c1-debug.apk. These APK files are the Android apps you built in Step 3.

Part II: Install Android app(s) on a tablet

• The Android tablet used must support **Android version 5.1 (Lollipop)**. Most (>90%) tablets available today support this version of Android.
• You must also enable the **Install Unknown Apps** permission, as we'll be installing the apps outside of the Google Play store. To do this, follow these instructions: https://developer.android.com/distribute/marketing-tools/alternative-distribution#unknown-sources
• Now the easiest way to transfer the apps to the tablet is to attach the three. apk files from Step 4 to an email message and access that email message from the tablet. When you open the email on the tablet, there should be an option to install the. apk file from the email attachment.
  ○ If your email provider blocks sending emails with an. apk attachment, Google Drive also works. Alternatively, you can drag and drop the. apk file from your computer to the tablet's file system using a USB cable.

At this point, the app(s) should be installed on the tablet, and show up on the home screen.

### Data export

Data from the task are saved on the tablet as a JSON file. A short python script (called irt_data_to_csv.py) converts the JSON format to a CSV file once the data has been copied to a computer with python installed. This script is also available at: https://osf.io/hc2ne/. To run the script, save it to the same location as the JSON file, and run the following on the command line:

$ python irt_data_to_csv.py [./path/to/irt_data] [./path/to/output_csv_file]

where "/path/to/irt_data" is the filepath of the data from the app, and "/path/to/output_csv_file" is the location you'd like to save the .csv file to.

## Funding

This work was supported in part by grants from the TKF Foundation, the John Templeton Foundation (University of Chicago Center for Practical Wisdom and the Virtue, Happiness, and Meaning of Life Scholars Group), and the National Science Foundation [Grant BCS-1632445]. Partial support comes from the international research fellowship grant from the Swedish Research Council [reference no 2015-00190].
